# Dietary Strategies to Improve Exercise Performance by Modulating the Gut Microbiota

**DOI:** 10.3390/foods13111680

**Published:** 2024-05-27

**Authors:** Li Zhang, Haoyu Li, Zheyi Song, Yanan Liu, Xin Zhang

**Affiliations:** 1 Department of Physical Education, China University of Mining and Technology, Beijing 100083, China; zhangli304036@126.com (L.Z.); 17718577057@163.com (H.L.); 2 Department of Food Science and Engineering, Ningbo University, Ningbo 315211, China; szy719792213@163.com (Z.S.);

**Keywords:** gut microbiota, exercise, diet, probiotics

## Abstract

Numerous research studies have shown that moderate physical exercise exerts positive effects on gastrointestinal tract health and increases the variety and relative number of beneficial microorganisms in the intestinal microbiota. Increasingly, studies have shown that the gut microbiota is critical for energy metabolism, immunological response, oxidative stress, skeletal muscle metabolism, and the regulation of the neuroendocrine system, which are significant for the physiological function of exercise. Dietary modulation targeting the gut microbiota is an effective prescription for improving exercise performance and alleviating exercise fatigue. This article discusses the connection between exercise and the makeup of the gut microbiota, as well as the detrimental effects of excessive exercise on gut health. Herein, we elaborate on the possible mechanism of the gut microbiota in improving exercise performance, which involves enhancing skeletal muscle function, reducing oxidative stress, and regulating the neuroendocrine system. The effects of dietary nutrition strategies and probiotic supplementation on exercise from the perspective of the gut microbiota are also discussed in this paper. A deeper understanding of the potential mechanism by which the gut microbiota exerts positive effects on exercise and dietary nutrition recommendations targeting the gut microbiota is significant for improving exercise performance. However, further investigation is required to fully comprehend the intricate mechanisms at work.

## 1. Introduction

The term “gut microbiota” refers to the broad community of microorganisms that live in the human gut. This community is made up of between 10 and 100 trillion microorganisms, including bacteria, fungi, protozoa, and viruses [1]. One hundred fifty times more genetic material is found in the gut microbiome than in the human genome, and over 100 different bacterial species are present [2]. Studies classified 12 different phyla of the microbiota community; approximately 90% belonged to *Firmicutes*, *Bacteroidetes*, *Proteobacteria*, and *Actinobacteria* [3]. Microorganisms in the microbiota restrict and depend on each other, playing a fundamental role in host homeostasis, including metabolic processes, immunity, and neuromodulation of the human body [4]. In addition, the resident microbiota plays a part in numerous biological processes, ranging from nutrition metabolism, short-chain fatty acid (SCFA) fermentation, and vitamin synthesis to regulation of brain function [5]. The gut microbiota significantly affects the immune system, nutrition, and metabolic function of the host and may be regulated by external environmental conditions, including medications, diseases, diet, and lifestyle [6]. Maintaining the ecological stability of the gut microbiota is extremely critical for physical wellness. As an external environmental factor, physical activity is also considered to impact the homeostasis and physiological changes of the gut microbiota. Cross-sectional studies have shown that a physically active lifestyle or moderate exercise seems to increase gut microbiota diversity and promote beneficial microbiota growth [7,8].

Recently, growing evidence have revealed that the gut microbiota has a positive correlation with exercise outcomes, and relevant observational experiments and mechanism studies have received wide attention from researchers. The gut microbiota impacts the host’s nutritional and metabolic status, promotes the breakdown and absorption of foods, and helps the body acquire and store energy, processes that are closely related to energy utilization during exercise [9]. A fluid range of research indicates that the gut microbiota affects inflammation response, stress resilience, nerve function, and spiritual health, all factors that are important for exercise performance [10,11]. Microbiota composition and the profiles of butyrate were shown to be significantly altered between active and sedentary rats in rodent tests [12]. Similar results have also been reported in humans, indicating that exercise and physical activity increase gut microbial diversity [7,13]. Moreover, changes in gut microbial composition were closely connected to metabolic activities [14]. The relative abundance of gut bacteria, which are responsible for breaking down lactic acid, a metabolic product of intense exercise, was shown to be enhanced in marathon runners [15]. Additionally, gut microbiota metabolites can improve exercise-induced gastrointestinal tract disorders like diarrhea and intestinal barrier dysfunction [16]. Nonetheless, current studies focus on observational experiments and are influenced by a variety of factors. Research on the connection between gut microbiota composition or microbial physiological function and exercise is still lacking.

Dietary nutrition can directly affect microbial communities and metabolites; therefore, regulating the gut microbiome through the implementation of dietary strategies is an effective means to enhance exercise performance [17]. Food type, quality, and origin modify the structure and function of our gut microorganisms, which, in turn, impact host–microbe interactions [18]. The gut microbiota utilizes energy, nutrients, micronutrients, and dietary fiber that can produce beneficial metabolites, such as SCFAs and neurotransmitters [19]. It has been demonstrated that SCFAs can promote the absorption and oxidation of lipids and glucose and encourage the efficiency of glycogen synthesis [20]. Supplementation with probiotics (live bacteria that can play a positive role in the health of the host after appropriate intake) and prebiotics (substances that can be selectively utilized by host microorganisms and have beneficial effects on host health) is a common approach to regulating the gut microbiota. They could reshape the gut microbiota’s structure, increase microbiota diversity, and encourage more health-promoting species [21]. Moreover, probiotics may have positive effects on mental responses, thus alleviating psychological stress during exercise. Probiotic supplementation was shown to improve cognitive function and overall mental state, with lower levels of depression and anxiety symptoms during straining [22]. Therefore, dietary interventions that target the gut microbiota provide an innovative means of improving exercise performance. 

In this article, we first summarize the research progress with respect to changes in the gut microbiota induced by exercise and the negative effects of excessive exercise on gut health. Then, we propose three specific mechanisms by which the gut microbiota influences exercise performance, including skeletal muscle function, oxidative stress, and the neuroendocrine system. Finally, we provide dietary recommendations during exercise based on the gut microbiota, including with respect to carbohydrates, protein, and fat, as well as an overview of the effects of probiotics and synbiotics on exercise performance, which may guide the dietary regimen for exercise.

## 2. Impacts of Exercise on the Gut

Physical exercise is an important means to promote health and chronic disease management and can exert a positive impact on the body through energy metabolism, inflammatory response, and oxidative stress [23]. Moreover, moderate exercise exerts positive effects on gut physiology health, such as by promoting gastrointestinal peristalsis to relieve constipation and accelerating blood circulation in the gastrointestinal tract [24]. Studies have confirmed that exercise can affect the diversity, composition, and relative abundance of the gut microbiota [7,25]. Furthermore, there are reciprocal effects between the gut microbiota and exercise. Exercise-related alterations in the gut microbiota and microbial metabolite variations have also been reported, indicating that the gut microbiota may influence the effects of exercise [12,26]. We provide a summary of the connections between exercise and gut health below.

### 2.1. Exercise Induces Changes in the Gut Microbiota

Since the gut microbiota plays a major role in many chronic illnesses, exercise may have positive effects by modifying the gut microbiota. In particular, those with metabolic problems can benefit from exercise by maintaining positive regulation of their gut microbiome. Exercise training during diet-induced obesity in mice enhanced the α-diversity and *Bacteroidetes*/*Firmicutes* ratio of the distal gut and fecal microbiota [27]. Compared with no-exercise participants, overweight/obese prediabetes males have altered microbiota profiles with increased SCFA biosynthesis and branched-chain amino acid catabolism. The changes in the gut microbiota induced by exercise were positively correlated with improvements in glucose homeostasis and insulin sensitivity [28]. In addition to obese individuals, exercise also exerts a positive effect on the gut microbiota of sedentary individuals. A study of women showed differences in gut microbiota between active and sedentary women. Beneficial bacterial species were increased in women with exercise, including *Akkermansia muciniphila* and butyrate-producing bacterial genera *Faecalibacterium prausnitzii* and *Roseburia hominis*, showing that an active lifestyle is positively correlated with the gut microbiota [29]. Similarly, a non-randomized comparative trial of sedentary women (≥65 years) conducted by Morita et al. showed that aerobic activity was dramatically raised the relative abundance of *Bacteroides* in healthy older women. Meanwhile, participants who increased their brisk-walking duration by more than 20 min had a significant rise in the relative abundance of intestinal *Bacteroides* [24]. Similarly, cardiorespiratory fitness training elevated α-diversity and boosted butyrate-producing numbers (*Roseburia*, *Lachnospiraceae*, *Clostridiales*, and *Erysipelotrichaceae*), which contributed to increases in butyrate levels [30]. Furthermore, compared to individuals with a sedentary lifestyle, athletes were observed to have higher α-diversity. A more enriched profile of SCFAs and increased metabolic pathways (amino acid biosynthesis, vitamin biosynthesis, and lipid biosynthesis) were also exhibited, which help in muscle turnover [31]. In particular, gut microbial diversity was increased in male professional rugby players. Athletes with low BMI had a markedly higher abundance of *Akkermansia muciniphilla* compared with the high-BMI group. Moreover, microbiota diversity showed a positive connection with protein intake and creatine kinase levels [7]. 

Changes in the gut microbiota’s diversity, composition, and abundance during exercise may depend on the subject, exercise style, duration, and exercise intensity. The gut microbiota of athletes varies depending on the kind of sport they play, according to a recent multi-cohort research study [32]. Among marathon runners, *Veillonella atypica* was found in higher relative abundance. Subsequently, a significant increase in exhausting treadmill runtime was observed in mice injected with this strain. Lactate may be used by *Veillonella* as a carbon source; further analysis found that the metabolic pathways of converse lactate into propionate were enriched in athletes [15]. Data from an exploratory study showed that the gut microbiomes of cyclists were divided into two groups, namely those with high *Prevotella* and *Bacteroides* levels and those with a mixture of several taxa, including *Bacteroides*, *Eubacterium*, *Ruminococcus*, *Prevotella*, and *Akkermansia* [33]. As a methanogenic bacterium, *Methanobrevibacter smithii* can promote fermentation efficiency, thereby increasing the production of SCFAs, adenosine triphosphate (ATP), and other important compounds to encourage athletes’ performance. Unfortunately, the study did not have a diet control or a non-athlete control group [33,34]. The gut microbiota can also be affected by exercise intensity. A 6-month randomized controlled trial compared the effects of different intensities of exercise, namely moderate- or vigorous-intensity biking, on the gut microbiota. The results showed that all exercise groups had altered β-diversity compared with the control group, and the vigorous exercise group had a significant increase in α-diversity [35]. In an effort to better understand bacteria’s reaction to short variations in training volume, Hampton-Marcell et al. found a sharp decline in microbial diversity, the structural similarity of communities of bacteria was dramatically decreased, and the proportion of genera *Faecalibacterium* and *Coprococcus* was significantly reduced in training volume [36]. In addition, whether the psychological state of exercise (voluntary/forced exercise) affects the gut microbiota needs to be confirmed by more studies. A mouse model showed that neither voluntary nor forced moderate exercise result in significant differences in bacterial diversity in gut microbiome. However, machine learning could detect minute changes in the mouse microbiome in response to exercise and reflect microbial interactions [37]. Taken together, gut microbial composition and structure are subjected to the multiple factors of exercise, and variable conditions need to be controlled to reflect the changes and roles of microorganisms in it.

### 2.2. Negative Effects of Excessive Exercise on Gut Health

Although exercise is a health benefit, it may become detrimental when the physical activity is intense and with inadequate rest and nutritional conditions. Indeed, endurance athletes such as runners and triathletes are highly susceptible to gastrointestinal problems, such as nausea, heartburn, diarrhea, and gastrointestinal bleeding [38]. A study of 272 endurance runners showed that 96% of examined runners had gastrointestinal symptoms, most commonly flatulence, belching, and nausea [39]. Moreover, research found that excessively strained mice presented with immune and metabolism disturbances and had lower microbial diversity [40]. This triggered intestinal inflammation and improve the growth of *Ruminococcus gnavus*, *Oscillospira* spp., *Butyrivibrio* spp., and *Coprococcus* spp., with a decrease in *Turicibacter* spp. [16]. Similarly, a multiple-stressor military training environment led to elevated intestinal permeability and inflammation. The intestinal microbiota exhibits increased α-diversity and decreased abundance of dominant *Bacteroides* groups [41]. Moreover, increased oxidative stress and disturbed intestinal barrier function can affect the homeostasis of the gut microbiota. Marathon runners often suffer from digestive problems like gastrointestinal bleeding, occult bleeding, and nausea [40]. Ultra-endurance exercise was observed to be associated with a high incidence of gastrointestinal symptoms, which may be related to rises in serum lipopolysaccharide (LPS) levels and intestinal permeability after intense exercise [42]. Blood flow preferentially pools away from the intestines when body temperature increases, supplying oxygen to the muscles and brain. As a result, a disturbance of the intestinal barrier and a decrease in intestinal blood flow result in the release of LPS into the bloodstream [43,44]. Ischemia and oxidative damage could contribute to elevated key phosphorylation enzymes, which disrupt intestinal epithelial tight junction proteins [45]. LPS leakage triggers the secretion of many pro-inflammatory cytokines and may influence the gut microbiota and further aggravate chronic inflammation [46]. The abovementioned research studies revealed that attention should be paid to the physiological effects of intense exercise on the gut. Furthermore, additional research is required to ascertain whether the composition or metabolites of the gut microbiota may reflect and detect overtraining or incorrect and/or inadequate training.

## 3. The Mechanisms of the Gut Microbiota with Respect to Exercise Performance

**Figure 1:**
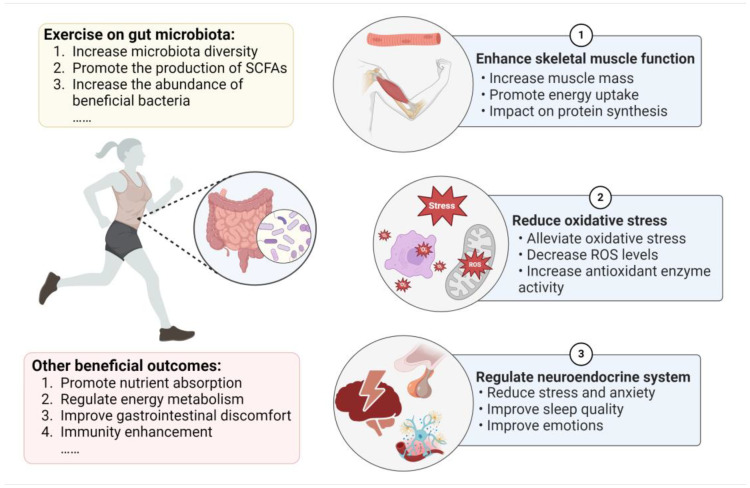
The effects of exercise on the gut microbiota and the mechanism of the microbiota’s role in improving exercise performance.

Numerous research studies have established that the microbiota plays a key role in energy metabolism, the intestinal mucosal barrier, immune regulation, and antioxidant effects [47]. The balance of the gut microbiota can reduce exercise fatigue by promoting lactate breakdown and enhancing mitochondrial function to help improve energy levels [8,48]. Additionally, it has been demonstrated that microbial composition is closely related to muscle glycogen content. In skeletal muscle, SCFAs may act as regulators to enhance fatty acid absorption and oxidation [49,50]. Recent studies have also reported that the gut microbiota communicates with the central nervous system, which influences stress, sleep, and mental health during exercise [51]. Therefore, the beneficial impacts of the gut microbiota on exercise are multifaceted, and we mainly focus on the mechanism of the intestinal microbiota in enhancing skeletal muscle function and reducing oxidative stress, in addition to the role of the neuroendocrine system in exercise performance (Figure 1).

### 3.1. Gut Microbiota Enhances Skeletal Muscle Function

Recently, a growing number of studies have explored significant biological links between skeletal muscle and the gut microbiota, indicating that muscle function is largely mediated by the gut microbiota either directly or indirectly [52]. Germ-free (GF) mice showed signs of muscular atrophy and reduced transcription genes linked to the growth of skeletal muscle. Conversely, GF mice who received a gut microbiota transplant from pathogen-free (SPF) mice showed improvements in their oxidative metabolic capacity, increased skeletal muscle mass, and decreased indicators of skeletal atrophy [53]. Furthermore, prior studies showed that shifts in the gut microbiota impacted muscle fiber characteristics and fiber type distribution in GF mice upon transplantation of the intestinal microbiota of obese Rongchang pigs [54]. It is commonly recognized that the phenotype, mass, and function of skeletal muscle depend on protein metabolism. Through the utilization of certain amino acids from diet and endogenous proteins, the gut microbiota can change the bioavailability of amino acids, which regulate the synthesis and breakdown of muscle protein [55]. The activation of the mammalian target of rapamycin (mTOR) is important for increasing muscle protein synthesis. Insulin-like growth factor-1 (IGF-1) is a major growth hormone influencing the maturation of bone, cartilage, and muscle [56]. IGF-1 expression was downregulated, and skeletal muscle mass was significantly reduced in mice with gut microbiome deletions [53]. In the mechanism, IGF-1 promoted skeletal muscle development via the phosphatidylinositol 3-kinase (PI3K)/protein kinase B (Akt)/mTOR pathway [57]. A study by Qi et al. revealed that GF piglets showed signs of muscle loss and muscle atrophy. The inactivation of the IGF-1/Akt/mTOR pathway caused muscle atrophy and autophagy. However, microbiota transplantation partially restored muscle growth and development, suggesting that skeletal muscle functions are mediated by the gut microbiota [58]. 

In addition, dysbiosis of the gut microbiota may decrease skeletal muscle glucose availability and glycogen content in muscles, thereby influencing energy metabolism during aerobic exercise [59]. Compared with normal mice, GF mice exhibited lower muscle glycogen levels [60]. Gut microbiota depletion reduces the ability of skeletal muscle to store glycogen and metabolize glucose, which hinders exercise capacity [61]. Glucagon-like peptide-1 (GLP-1) has a role in reducing glycemia and regulating insulin and glucagon secretion. Previous investigations have proven that GLP-1 can effectively elevate microvascular blood flow, thereby increasing skeletal muscle glucose transport [62]. A recent study suggested that overexpressing GLP-1 in skeletal muscle could enhance endurance capacity by increasing glycogen synthesis, glucose uptake, and the proportion of type I fibers, as well as mitochondrial biogenesis [63]. The results also verified that GLP-1 regulated skeletal muscle remodeling through AMP-activated protein kinase (AMPK), which is a cellular energy receptor of ATP consumption and regulates skeletal muscle metabolism [63]. 

SCFAs not only serve as an energy source but are also essential for improving normal skeletal muscle function and exercise performance. According to Okamoto et al., low-microbiota mice exhibited reductions in bacterial diversity and the amount of SCFAs, which resulted in a decrease in the mass of the tibialis anterior muscle and treadmill running time. Meanwhile, mice given antibiotics had decreased running ability, but SCFA acetate injection increased their capability for endurance exercise [64]. The production of SCFAs by the gut microbiota plays a positive role in promoting glucose metabolism in skeletal muscle. Nay et al. found that endurance running capacity and skeletal muscle contractile function were impaired in mice with antibiotics, noting the microbiota metabolite SCFAs are involved in the bioavailability of intramuscular fuels (such as glycogen and triacylglycerol) [60]. SCFA receptors are located in colonic enteroendocrine L cells producing and secreting GLP-1. Increased concentrations of SCFAs could increase GLP-1 production by stimulating L-cell SCFA receptors or by other means [65,66,67]. Moreover, SCFA can increase the AMP content that induces AMPK phosphorylation in myotubes and skeletal muscles [68]. Indeed, SCFAs such as acetic acid have been reported to improve fatty acid metabolism and glucose absorption in skeletal muscle cells in vitro by activating AMPK [69]. Moreover, SCFA can increase insulin sensitivity and promote glucose uptake and glycogen synthesis in skeletal muscle to increase skeletal muscle glycogen content [49]. These findings show the dominant effects of microbial SCFAs on exercise performance.

### 3.2. Gut Microbiota Reduces Oxidative Stress

The mitochondria is the main source of energy for the oxidization of lipids and carbohydrates to produce reactive oxygen species (ROS) and ATP. Two other major sources of ROS generation in the intestinal lumen are migratory neutrophils and intestinal epithelial cells [70]. When performing aerobic activity, the demand for ATP increases in skeletal muscle, potentially overloading the mitochondria’s electron transport chains and producing superoxide [71]. Research has revealed that moderate exercise could increase antioxidant levels. For instance, exercise-induced ROS generation results in beneficial outcomes during regular physical activity, which can contribute to increased mitochondrial biogenesis and stimulate antioxidant enzymes. Mitochondrial ROS can activate antioxidant enzymes including manganese superoxide dismutase, catalase (CAT), and superoxide dismutase (SOD) [9], whereas intense exercise can induce excessive ROS formation. Further oxidative stress could potentially cause damage to lipids and protein peroxidation, which potentially disrupts the muscle cell membranes and influences muscle function [72,73]. Recent research revealed that excessive exercise training for 2 weeks led to higher levels of oxidative proteins and triggered high-intensity permeability and tight-junction damage. Inhibition of ROS production suppressed the elevation of intestinal permeability induced by exercise [74]. Moreover, pro-inflammatory cytokines such as IL-6, IL-1β, TNF-α, and NF-κB are also elevated during intense exercise [75]. 

The gut microbiota and microbial metabolites help alleviate exercise-induced oxidative stress and fatigue. In vitro studies have proven that SCFAs present antioxidative properties; for example, butyrate has the ability to alleviate oxidative stress and decrease ROS levels, as well as mitochondrial dysfunction, by activating the AMPK pathway [76]. The homeostasis of the gut microbiota also plays a critical part in antioxidant enzyme activity and oxidative stress levels [59]. According to animal research conducted by Hsu et al., SPF mice had substantially longer endurance swimming times than GF mice, and the serum levels of glutathione peroxidase and CAT activities were, likewise, significantly higher, which suggests that microorganisms may improve exercise fatigue by increasing the levels of antioxidant enzymes, thus improving exercise capacity [77]. Studies conducted by Mazani et al. showed that probiotic yogurt administration improved glutathione peroxidase in young females following exhaustive exercise [78]. Data from Huang et al. showed that *Lactobacillus plantarum* PS128 supplementation in triathletes can significantly alleviate oxidative stress (CAT, thioredoxin, and myeloperoxidase indices) and reduce pro-inflammatory cytokines after a triathlon [79]. Moreover, daily consumption of a mixture of probiotics based on *B. longum*, *L. casei*, and *L. rhamnosus* markedly reduced lipid-related and DNA-related oxidative stress biomarkers during high-intensity and long-duration physical exercise [80]. A study by Martarelli et al. revealed that supplementation with probiotic *Lactobacillus rhamnosus* IMC 501 and *Lactobacillus paracasei* IMC 502 boosted plasma antioxidant levels and neutralized the effects of ROS, exerting strong antioxidant activity in athletes during intense exercise [81]. In summary, the gut microbiota can reduce oxidative stress through various mechanisms, but specific bacterial groups have different effects on antioxidant enzymes. More research is required to fully comprehend how the gut microbiota regulates the redox balance in response to vigorous exercise.

### 3.3. Gut Microbiota Impacts the Neuroendocrine System

Athletes often suffer from stress, anxiety, mood disturbances, and sleep disorders when overtraining and on the eve of major competitions, which lead to fatigue and decreased athletic performance. It is believed that endurance exercises such as triathlon and long-distance running have a higher risk of stress [82]. The major stress system is mainly composed of the hypothalamus–pituitary–adrenal (HPA) and sympathoadrenal–medullary axes. These axes are activated by stress during exercise, which causes the production of corticotropin-releasing hormone (CRH) and the secretion of adrenocorticotropic hormone (ACTH) into the circulatory system and stimulates the secretion of cortisol [83]. Intense physical exercise that exceeds 60% of maximum oxygen absorption may stimulate the HPA axis and result in a spike in serum cortisol. Moreover, research on endurance athletes found exercise-induced stress and hormone release, suggesting that 60–80% of athletes in the early stages of chronic stress have increased CRH-activated ACTH responses [16]. Furthermore, synchronic (concomitant) sleep disturbance is accompanied by a substantially activated HPA axis, a diachronic decrease in total sleep time, and slow-wave sleep [84]. In particular, sleep helps the body recover from exhaustion by healing processes and recovering energy, with critical effects on physiology and neuron biology [85]. Sleep may also chronically change glucose metabolism and neuroendocrine function, ultimately negatively influencing athletes’ nutritional and endocrine condition [86]. Furthermore, physical fatigue in overtraining is associated with the brain’s serotonin (5-HT) level, increased 5-HT-effect drowsiness, and central fatigue, and a low level of 5-HT in the brain could result in depression-like behavior and mood disorders [87]. 

Growing evidence has demonstrated that the gut microbiota can communicate with the brain along the gut–brain axis in direct and indirect ways (neural pathways, endocrine pathways, and immune pathways) and subsequently influence mood and behavior [88]. In the human body, the gut microbiota helps regulate the generation of 5-HT and 5-hydroxytryptophan (the precursor of 5-HT, which can pass the blood–brain barrier and take part in the brain’s production of 5-HT). The enterochromaffin cells in the intestinal mucosa are responsible for producing about 90% of the 5-HT in the body [89]. Exercise-induced central fatigue symptoms are related to the 5-HTeric system. Research has indicated that acute exercise leads to a considerable increase in 5-HT production and metabolism in the hypothalamus and brain stem [90]. Overtraining causes unbound blood tryptophan levels to rise and reduces branched-chain amino acids in the blood, thereby producing a large amount of 5-HT in the brain, which leads to long-term fatigue [91]. Moreover, Vargas and Marino proposed that immune cytokines released during acute exercise might signal the brain, leading to neuroinflammation and the feeling of fatigue [92]. Prolonged strenuous exercise may induce elevated circulating LPS levels, and an increase in intestinal permeability may provoke neuroinflammatory responses and the perception of fatigue [93]. According to several studies, high levels of LPS and pro-inflammatory cytokines have revealed a connection between anxiety and depression behaviors [94]. However, present experimental data lack information on the gut microbiota’s effect on neurotransmitters and neuroinflammation during exercise. Several studies have explored the role of probiotic supplementation in exerting positive impacts in terms of alleviating stress, mental status, and sleep quality for athletes. Bacterium *Bifidobacterium* and *Lactobacillus* strains could significantly reduce stress and anxiety behaviors, in addition to possessing antidepressant-like potential [95]. Randomized controlled trials have shown that probiotic supplementation offers a small and significant positive effect in tests with aerobic metabolism predominance in comparison with placebo. For example, diving athletes who were administered a mixed probiotic yogurt containing *Bifidobacterium animalis* ssp. *lactis* BB-12 (1 × 10^9^ CFU/100 g) felt less pressure [96]. A single strain of *Bifidobacterium animalis* ssp. *lactis* BB-12 also improved emotion and facilitated the clearance of psychological fatigue after composition compared with the control group [97]. A randomized placebo-controlled study showed that consuming a probiotic drink containing *Lactobacillus casei* at a dose of 3 × 10^10^ CFU alleviates competitive anxiety and stress for athletes, indicating that probiotics could help athletes improve their mental situation and physical performance [98]. Additionally, 17 weeks of probiotic combination (*Lactobacillus*, *Bifidobacterium*, and *Streptococcus*) supplementation enhanced rugby players’ subjective sleep quality and helped them sense less muscle soreness compared to a placebo [99]. Similarly, a triple-blinded randomized controlled study demonstrated that supplementation with a synbiotic containing probiotic strains for 4 weeks resulted in enhanced measures of anxiety, stress, and sleep quality by increasing dopamine and the CRH concentration in soccer players [100]. These results could be influenced by the type of sport, strain type, dosage, supplementation duration, and participant characteristics (e.g., age, sex, health status, and nutrition). Further research could also mechanistically elucidate how the gut microbiota improves exercise performance by affecting the gut–brain axis.

## 4. Dietary Recommendations for Targeting of the Gut Microbiota

**Figure 2:**
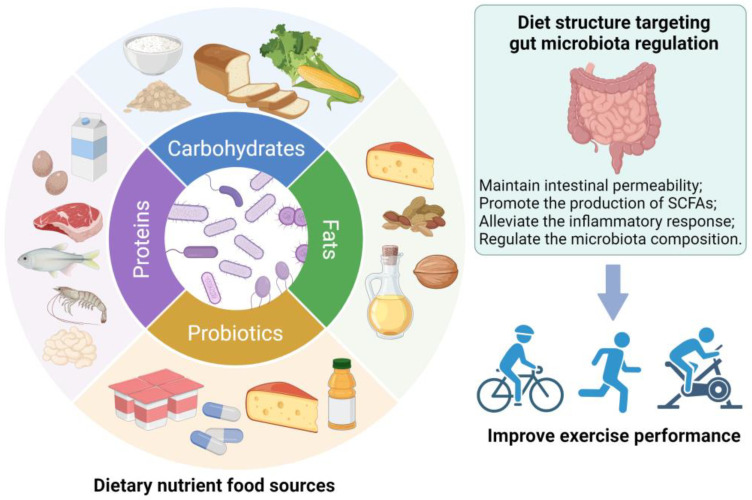
Diet structure targeting gut microbiota regulation has positive effects in terms of improving exercise performance.

In light of diet strongly influencing the gut microbiota, nutritional regulation targets the gut microbiota according to the overall nutritional status, exercise intensity, and physiological condition of athletes can improve exercise performance (Figure 2). Alternatively, considerable evidence has accumulated to show that probiotics have the potential to boost the physical health and performance of athletes [17]. Next, we will discuss dietary nutrition strategies focused on modulating the gut microbiota.

### 4.1. Dietary Strategies

#### 4.1.1. Carbohydrates

Unquestionably, consuming sufficient carbohydrates is necessary for training and exceptional exercise performance. During prolonged activity, depleted muscle and liver glycogen is restored by consuming carbohydrates, which helps reduce elevated levels of stress hormones such as cortisol, improves physical performance, reduces the perception of fatigue, and improves mood state [101]. Athletes who train more than 2 h daily must take in 7–12 g/kg of carbohydrates per day [102]. Continuously insufficient amounts of carbohydrates may impair the immune system, cause nervous damage, and limit the body’s ability to respond to heavy physical demands [102]. The gut microbiota fermentates non-digestible carbohydrates through a variety of hydrolytic enzymes to produce SCFAs. Dietary strategies for stimulating SCFA production in athletes may be beneficial, in part, to improve exercise performance, since SCFAs are associated with increased muscle function and glycogen [60]. Baxter et al. demonstrated that potato starch greatly increased total SCFAs including butyrate, with an increase in the relative abundance of *Bifidobacteria* [103]. Intake of whole grains (40 g/d of fiber) increased the abundance of SCFA-producing genera *Lanchnospira* and *Roseburia* compared to refined grain [104]. It is also recommended that athletes supplement their diets with oligosaccharides such as galacto-oligosaccharides and fructose oligosaccharides, which can promote beneficial intestinal metabolism by increasing the intake of plant foods (such as cereals, legumes, vegetables, and fruits) [105]. Furthermore, increasing fiber such as resistant starch can reduce the negative effects caused by high protein consumption [106]. According to a study conducted on animals by Donatto et al., feed rich in oat-bran β-glucan enhanced the storage of glycogen in the muscles and liver, as well as SCFA production, and reduced the inflammatory response after exercise [107]. In a randomized trial, the effects of partly hydrolyzed guar-gum fiber on the gut microbiota of healthy male athletes were shown to be associated with lower levels of the *Prevotella*, *Bacteroidetes*, and *Clostridium* cluster; increased levels of *Actinobacteria*; and reductions in diarrhea and fecal excretory sensation [108]. However, to minimize gastrointestinal discomfort, athletes should ingest small amounts of fiber before exercise. Supplementing moderate amounts of simple carbohydrates (such as glucose, sucrose, and fructose) during exercise can alleviate exercise fatigue [17]. The literature suggests that a close ratio of glucose and fructose intake can not only improve the carbohydrate oxidation rate but also improve gastrointestinal comfort [109]. Lactose can be considered in dietary regimens before or during exercise, since it is not as readily oxidized, helps in glycogen restoration, and may encourage the growth of *Bifidobacteria* and *Lactobacilli* [110]. In conclusion, athletes are suggested to increase dietary fiber intake daily and decrease the intake of high sugars and refined carbohydrates. A supply of simple carbohydrates is also suggested to maintain glucose before exercise to maximize carbohydrate absorption, and lower levels of fiber intake are suggested to decrease the occurrence of gastrointestinal symptoms [17].

#### 4.1.2. Protein

In general, physical exercise requires a large amount of protein. Admittedly, protein is essential for muscle synthesis and metabolism, especially for those athletes who aim to gain muscle mass and strength [111]. A recommendation of the American College of Sports Medicine suggests intakes ranging from 1.2 to 2.0 g/kg/day, whether performing aerobic or resistance exercise [112]. A high-protein diet (HPD) promotes muscle mass during strength training and alleviates the negative effects of highly eccentric strength training, such as soreness and fluctuations in muscle protein concentration [113]. Adequate protein intake also supports skeletal muscle metabolism, immune function, and intestinal mucosal integrity [114]. In the meanwhile, colonocytes can obtain free amino acids as an alternate energy source through the degradation of proteins [115]. However, an HPD may increase the abundance of proteolytic microbes and affect the composition and balance of the gut microbiota [111]. For instance, endurance training athletes who consumed 20 g of protein per day for 10 weeks had a 3.8-fold reduction in levels of the beneficial bacteria *Roseburia*, *Blautia*, and *Bifidobacterium longum* and an increased abundance of *Bacteroidetes* phylum [116]. Amino acid metabolites of the gut microbiota can be divided into sulfur-containing amino acids, aromatic amino acids, and tryptophan metabolites, including SCFAs, ammonia, sulfides, and indolic and phenolic compounds. Excessive protein intake will lead to harmful metabolites and damage intestinal epithelium integrity, leading to intestinal inflammation [117]. An animal study revealed that rats fed an HPD exhibited increased *Streptococcus*, *Escherichia/Shigella*, *Enterococcus*, and sulfate-reducing bacteria but reduced beneficial bacteria *Ruminococcus*, *Faecalibacterium prausnitzii*, and *Akkermansia* in the colon as compared to a normal-protein diet [118]. In line with these results, rats fed an HPD had greater amounts of *Escherichia coli* and decreased SCFA-producing bacteria *Akkermansia muciniphila*, *Bifidobacterium*, *Prevotella*, *Ruminococcus bromii*, and *Roseburia/Eubacterium rectale* [119]. An observational study showed that bodybuilders on an HPD had greater amounts of *Faecalibacterium, Sutterella, Clostridium, Haemophilus*, and *Eisenbergiella* and decreased *Bifidobacterium* and *Parasutterella* [120]. Moreover, consideration needs to be given to the impact of various protein sources on gut health. Dietary proteins from processed meat or red meat are a major source of L-carnitine and choline. These substances can be transformed by the gut microbiota to produce trimethylamine (TMA) and be oxidized to trimethylamine N-oxide (TMAO), an organic compound linked to an increased risk of atherothrombotic cardiovascular disease [115]. Thus, adequate protein intake should be ensured during exercise, and attention should be paid to the impact of dietary protein on the gut microbiota.

#### 4.1.3. Fat

The oxidation of fats provides energy for athletes. Especially during endurance exercise, fat metabolism helps to improve exercise performance. Different exercise types, training levels, and physical conditions affect the body’s requirement for fat [17]. In a study of bodybuilders, the relative abundance of *Sutterella* was found to be positively correlated with fat intake, but *Bifidobacterium* exhibited a negative correlation [120]. Murtaza et al. revealed that a ketogenic diet raised the relative abundance of *Bacteroides* and *Dorea* and resulted in a decrease in *Faecalibacterium*. Fat oxidation was substantially inversely correlated with the relative abundance of *Bacteroides*. These results may help explain the effects of drastic shifts in diet patterns on the gut microbiota of performance walkers undertaking intensified training [121]. The different types of dietary fats may be associated with varying impacts on the gut microbiota. Consuming large amounts of fat and saturated fatty acids has a detrimental effect on the richness and diversity of the microbiota; for example, a palm oil-based diet increased the ratio of *Firmicutes* to *Bacteroidetes* [115]. Omega-3 polyunsaturated fatty acids have the ability to boost the amount and population of beneficial bacteria, notably *Bifidobacterium*, *Lactobacilli*, and *Akkermansia*. It also inhibits the production of pro-inflammatory mediators, increases the concentration of SCFAs, and maintains the healthy balance of gut microbiota [122]. Furthermore, the gut microbiota mediates bile acid metabolism (changes the bile acid structure and composition) and can promote muscle growth and strengthen muscles [123]. Overall, one of the key factors of lipid supplementation during exercise is the relationship between dietary fats and the gut microbiota.

### 4.2. Probiotics and Synbiotics

The healthy and beneficial effects of probiotic supplementation on exercise have prompted increasing interest. Prolonged and physically demanding training and composition can lead to respiratory infection, gastrointestinal diseases, inflammatory disorders, immunosuppression, oxidative stress, anxiety, and fatigue [124]. As we mentioned above, strenuous exercise triggers increased intestinal mucosal permeability and induces inflammatory responses. In the past decade, research on the effects of probiotics on exercise and the types of nutritional supplements containing probiotics has increased significantly. We summarize the application of probiotic supplementation in exercise in recent years in Table 1. The literature shows that probiotics exert positive effects on athletes—mainly *Lactobacillus* and *Bifidobacterium* species [125]. Long-term high-intensity exercise may lead to immunosuppression and increase the risk of upper respiratory tract infection (URTI). Numerous studies have found probiotic supplementation to be beneficial for the frequency, intensity, and duration of URTI. In a recent study by Tavares-Silva et al., employing multi-strain probiotics supplements lowered the frequency and severity of URTI [126]. Additionally, the intake of capsules containing *Lactobacillus helveticus* shortened episode durations and decreased the frequency of URTI [127]. Triathletes supplementing with *Lactobacillus plantarum* PS128 showed microbiota reconstruction and increased SCFA contents, as well as increased homeostasis muscle energy as a result of increased plasma-branched amino acids. Such supplementation can also significantly alleviate oxidative stress, decrease pro-inflammatory cytokines, and increase anti-inflammation cytokines [79,128]. Moreover, the gut microbiota could impact gut–brain-axis functions like the synthesis of neurotrophic factors or transmitters, affecting neurodevelopment, cognition, and behavior via multiple pathways. Probiotic consumption was found to improve athletes’ mental health in several studies; nevertheless, only a few studies have examined the exact mechanism by which probiotic supplementation affects athletes’ mental and mood status. Probiotic consumption prevented exercise-induced decreases in tryptophan levels and improved the kynurenine/tryptophan ratio [129]. A double-blind randomized controlled trial involving elite male rugby athletes showed decreased muscle soreness and improved sleep quantity in the probiotic group [99]. Probiotics also improve nutrient absorption and utilization and energy metabolism and increase muscle mass. A meta-analysis showed that *Bifidobacterium* supplementation can enhance muscle mass and muscle strength [130]. A recent study of mice revealed that mice administered *Lactobacillus salivarius subspecies salicinius* (SA-03), separate from Olympic weightlifting athletes, exhibited significantly improved muscle strength and endurance performance. This was due to increased glycogen storage in the liver and muscles and decreased levels of lactate, blood urea nitrogen, ammonia, and creatine kinase following exercise [131]. In order to improve circulatory lactic acid catabolism following administration to the gut, Yang et al. modified probiotic *Bacillus subtilis* strains to produce lactate oxidase. Treatment of mice with engineered *B.subtilis* administered in the gastrointestinal tract has the potential to reduce serum lactic acid levels without impacting the normal composition of the gut microbiota, inflammatory response, and liver enzymes [132].

Synbiotics is a combination of synergistically acting probiotics and prebiotics with the aim of improving the survival of probiotic microorganisms in the gastrointestinal tract [133]. A previous double-blind controlled trial conducted over 21 days of exercise training showed that synbiotic supplementation (*Lactobacillus paracasei* 431, *Bifidobacterium animalis* ssp. *lactis* BB-12, *L. acidophilus* LA-5, *L. rhamnosus* LGG, raftiline, raftilose, lactoferrin, immunoglobulins, and acacia gum) was associated with a smaller increase in the serum IL-16 concentration but was associated with no substantial difference in fecal SCFA concentrations, mucosal immunity, or gastrointestinal tract permeability in comparison to the acacia gum group [134]. In contrast, a study compared the effects of long-term (6 weeks) prebiotic and synbiotic supplementation on the immune function of male football players after daily high-intensity training and a one-time strenuous exercise, finding that supplementation with synbiotics is better than with prebiotics at improving immune function by reducing the incidence of URTI and increasing the SIgA level. Results also showed that synbiotics could reverse the detrimental outcomes of one-time strenuous exercise [135]. Moreover, a triple-blinded study conducted by Quero et al. revealed that professional soccer players administered synbiotics (*Bifidobacterium lactis* CBP001010, *Lactobacillus rhamnosus* CNCM I-4036, *Bifidobacterium longum* ES1, and fructo-oligosaccharides) exhibited markedly improved anxiety, stress, and sleep quality [100]. These results suggest that the different effects of synbiotics may be correlated with the frequency and intensity of exercise, as well as the duration of the experiment.

The effects of probiotic supplementation vary depending on the individual’s diet structure; the physiological state of the host; microbiota composition; and the strain, dose, and duration of the probiotic. Due to the limited number of research studies on the impacts of probiotic supplementation on athletes’ physical function, relatively small samples and distinct training protocols have failed to specifically confirm the beneficial effects of probiotics. More research is currently focused on the use of combined probiotics in athletes. It would be interesting to further explore the effects of a single probiotic, namely *Bifidobacterium animalis* ssp. *lactis* BB-12, on exercise [96,97]. Furthermore, the longest exercise intervention available now persists for six months [35], and further research is needed to determine the effects of long-term exercise training when combined with probiotic application. It is vital to determine whether probiotic/synbiotic supplementation has an impact on the results because most probiotic supplementation studies do not assess the alteration of the intestinal microbiota. Moreover, the study of exercise and the gut microbiota cannot be separated from diet; in particular, the intake of prebiotics and dietary fiber needs to be considered in experimental design and analysis [17]. Further research is needed to combine exercise types and intensity, external environmental factors (lifestyle), accurate nutritional composition, and the use of metabolomic and proteomic technology to determine the type, concentration, and dose of probiotics that can improve exercise performance.

**Table 1:** The application of probiotic supplementation in exercise in recent years.

Intervention	Exercise Type	Study Model	Duration	Results	Reference
*Veillonella atypica*	Exhaustive treadmill runtime	Mice	2 days	Blood SCFAs concentration ↑ Lactate utilization ↑ Treadmill performance ↑ The Cori cycle ↑	Scheiman et al., 2019 [15]
*Lactobacillus plantarum PS128*	Triathlon championship	Triathletes	3 weeks	TNF-α, IFN-γ, IL-8, and IL-6 cytokine levels ↓ IL-10 cytokines levels ↑ Oxidative stress ↓ Thioredoxin and component 5a ↑ Free amino acid content ↑	Huang et al., 2019 [79]
Inactivated *Bacillus coagulans*	A self-defense course	Male soldiers	2 weeks	IL-10 and IFN-γ concentrations ↑ Vertical jump power ↑ Lower-body power and short-distance speed ↑	Hoffman et al., 2019 [136]
*Lactobacillus plantarum* PS128	Usual training	Triathletes	4 weeks	Acetic acid, propionic acid, and butyric acid ↑ Endurance capacity ↑ No effect on VO2max	Huang et al., 2020 [128]
*Bifidobacterium longum subsp. longum* Olympic No. 1 (OLP-01)	Usual training	Well-trained runners	5 weeks	12-min Cooper’s test running distance ↑ No change in the abundance of the gut microbiota.	Lin et al., 2020 [137]
*Lactobacillus salivarius* (SA-03)	Swimming exercise endurance test	Mice	4 weeks	Average exhaustive swim time ↑ Muscle strength and endurance performance ↑ Hepatic and muscular glycogen storage ↑ Serum lactate, blood urea nitrogen, ammonia, and creatine kinase levels ↓	Lee et al., 2020 [131]
Commercially available probiotic (PRO)	Usual training	Male elite cyclists	90 days	The incidence and severity of gastrointestinal tract symptoms ↓ No significant changes in VO2max, mean levels of IL-6, or TNF-α.	Pugh et al., 2020 [138]
*Lactobacillus casei*	Usual training	Male badminton players	6 weeks	Anxiety and stress levels ↓ Aerobic capacity ↑ No significant difference in energy intake and macronutrients.	Salleh et al., 2021 [98]
Probiotic blend	Usual training	Male elite cyclists	6 weeks	Incidence of gastrointestinal Symptoms ↓ No significant changes in IL-6 and TNF-α levels.	Schreiber et al., 2021 [139]
Probiotic (Ultrabiotic 60TM) and *Saccharomyces boulardii*	During competition season	Elite male rugby athletes	17 weeks	Muscle soreness and leg heaviness scores ↓ Sleep quantity, quality, and motivation scores ↑	Harnett et al., 2021 [99]
Probiotic (SANPROBI BARRIER)	During a marathon	Runners	3 months	Incidence of constipation ↓ Sodium, potassium, iron concentration, and HDL cholesterol ↑ LDL cholesterol and triglyceride concentration ↓	Smarkusz-Zarzecka et al., 2022 [140]
Yogurt with *Bifidobacterium animalis* ssp. lactis BB-12	Usual training	Female taekwondo athletes	8 weeks	L-arginine biosynthesis I, fatty acid biosynthesis and oxidation, and L-isoleucine biosynthesis III pathways ↑ Tyrosine degradation I ↓	Zhu et al., 2023 [132]
Heat-killed *Lactiplantibacillus plantarum* TWK10	Fixed-intensity exercise challenge	Healthy males	6 weeks	Exercise endurance time ↑ Muscle weight and muscle strength ↑ Serum lactate and ammonia levels during exercise ↓	Cheng et al., 2023 [141]

“↑”: Increase, “↓”: Decrease.

## 5. Conclusions

Most research has demonstrated a strong connection between the gut microbiota and physical exercise. Exercise potentially affects the diversity and composition of the gut microbiota, which, in turn, have impacts on exercise performance. The gut microbiota differs according to the type, intensity, and duration of exercise. Moderate exercise can promote the production of SCFAs and increase the abundance of beneficial bacteria, while excessive exercise can cause gastrointestinal discomfort, increase intestinal permeability, and provoke inflammatory responses. In addition, the gut microbiota can improve exercise performance by improving nutrient absorption, regulating energy metabolism, relieving fatigue, boosting skeletal muscle function, reducing oxidative stress, and regulating the neuroendocrine system. Additional research is required to examine the impacts of the gut microbiota on neurological and mental states through the gut–brain axis during physical exercise. From the standpoint of gut microbiota regulation, diet may be combined with exercise and dietary nutrition supplementation to enhance exercise performance. Currently, the majority of studies merely investigate the connection between the gut microbiota and exercise; further research is required to better understand the underlying mechanism to specify targeted intervention policies. Meanwhile, more extensive and varied sample populations are required to determine the specific probiotic strains that may induce desired responses in sporters.

## Data Availability

The original contributions presented in the study are included in the article, further inquiries can be directed to the corresponding author.
